# The developmental impact of sex chromosome trisomies on emerging executive functions in young children: Evidence from neurocognitive tests and daily life skills

**DOI:** 10.1111/gbb.12811

**Published:** 2022-05-18

**Authors:** Kimberly C. Kuiper, Hanna Swaab, Nicole Tartaglia, Griet van Buggenhout, Caroline Wouters, Sophie van Rijn

**Affiliations:** ^1^ Clinical Neurodevelopmental Sciences Leiden University Leiden The Netherlands; ^2^ Leiden Institute for Brain and Cognition Leiden The Netherlands; ^3^ eXtraordinarY Kids Clinic, Developmental Pediatrics Children's Hospital Colorado Aurora Colorado USA; ^4^ Department of Pediatrics University of Colorado School of Medicine Aurora Colorado USA; ^5^ Center for Human Genetics University Hospital Gasthuisberg Leuven Belgium; ^6^ Department of Human Genetics KU Leuven (University of Leuven) Leuven Belgium

**Keywords:** child development, child neuropsychology, executive function, genetic disorders, Klinefelter syndrome, trisomy X

## Abstract

Sex chromosomal trisomies (SCT) are associated with impairments in executive functions in school‐aged children, adolescents, and adults. However, knowledge on preschool development of executive functions is limited but greatly needed to guide early intervention. The current study examined emerging executive functions in young children with SCT. Participants were 72 SCT children and 70 population‐based controls, aged 3–7 years, who completed a neurocognitive assessment of both global executive function (MEFS) and verbal executive function skills (NEPSY Word Generation). Caregivers completed the Behavior Rating Inventory of Executive Function (BRIEF) questionnaire to capture real‐world behavioral manifestations of impairments in executive functions. Results showed that impairments were significantly more prevalent in SCT than in controls and already present from 3 years, specifically verbal executive functions and working memory. Broader more pronounced impairments were found in older children with SCT. Age was significantly related to executive functions, but specific domains showed different relations with age. For example, deficits in planning and organizing remained evident with older age in SCT whereas it declined with age in controls. Impairments in executive functions were present across different levels of intelligence. Already at an early age, impairments across executive functions should be considered part of the neurodevelopmental profile of SCT, which appear more prominent at later age. Future studies should investigate developmental pathways of executive functions in SCT, given its relevance in cognitive, social, and emotional development. Executive functions should be screened and monitored in children with SCT and could be an important target of preventive intervention.

## Graphical abstract

The current study shows that a sex chromosomal trisomy (SCT) in children is associated with an increased risk for problems with emerging executive functions, from as early as 3 years old, and that those problems appear more pronounced with increasing age. Furthermore, the vulnerability for difficulties with executive functions appears broader than the language domain alone, extending to other areas as well. Preschool executive functions are vital for school readiness, academic success, and psychological functioning, putting children with SCT at a developmental disadvantage compared to peers.

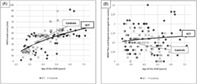

## 1 INTRODUCTION

With a high prevalence of 1–2 in 1000 births, sex chromosomal trisomies (SCT) are one of the most common chromosomal aneuploidies.^1^, ^2^ Karyotypes that result from SCT are 47, XXY (Klinefelter syndrome) and 47, XYY (XYY syndrome) in males and 47, XXX (Trisomy X syndrome) in females. Recent technological advances allow for safe and earlier screening for genetic syndromes and are expected to lead to an increase of the number of prenatally diagnosed children with SCT.^3^ This calls for more knowledge on the developmental impact of SCT which is needed to improve genetic counseling and clinical care for children with these conditions. Also, studying genetic conditions such as SCT from pregnancy on provides a unique opportunity to prospectively examine early neurocognitive development and its link to later developmental outcome.

Having an extra X or Y chromosome not only impacts physical development but also neurodevelopmental and psychological functioning.^4^ This is not surprising given the high density of genes on the sex chromosomes that are essential for brain development,^5^ putting children with SCT at increased risk for neurodevelopmental problems (i.e., impairments of growth and development of the brain, that may lead to differences in brain functioning and thus emotion and cognition amongst other domain). So far, neuroimaging studies have shown that brain architecture and functioning appears different in individuals with SCT compared to peers from the general population (XXY^6^; XXX/XXY/XYY^7^). Furthermore, underlying information processing difficulties are also found in individuals with SCT with a quarter of the group showing difficulties of clinical relevance (for a review see Reference [8]). Amongst other domains, impairments are found across executive functions while intellectual functioning is usually within the typical range (although at the lower end, particularly for verbal IQ) (XXY and XXX^9^; XXY^10^). Of relevance to the current study are studies showing neuroanatomical and functional differences in the (pre)frontal cortex in individuals with an extra X chromosome,^11^, ^12^ an area strongly involved in executive functions.^13^

The term *executive functions* (EF) refers to a set of interrelated cognitive skills essential to learn, cope, and manage daily life.^14^ Executive functions are responsible for purposeful, goal‐directed, and problem‐solving tasks and behavior. Several components can be identified, including attention, inhibition, monitoring, flexibility, working memory, planning, and fluency.^15^ Proper executive functions are crucial when it comes to positive childhood development: executive functions promote mental and physical health; predict success in school and in life; and support cognitive, social, and psychological development.^14^ On the other hand, impairments across executive functions are involved in many neurodevelopmental disorders, including attention deficit hyperactivity disorder (ADHD^16^), autism spectrum disorder (ASD^17^), and intellectual disabilities.^18^

Until now, studies that have examined executive functions in individuals with an extra X or Y chromosome showed, on average, reduced executive function performance compared to controls from the general population (for review see^19^). Children with SCT show more impairments across executive functions, including attention, inhibition, mental flexibility, working memory, and planning/problem solving.^10^, ^18^, ^20^, ^21^, ^22^, ^23^ In daily life, parents of children with an extra X chromosome (XXX and XXY) report difficulties in (sub)domains of behavioral regulation and meta‐cognition.^10^, ^18^ A substantial part of the SCT group shows significant executive function difficulties, that are present across studies and assessments. For example, 19% to 57% of children with SCT (both Dutch and American) show a clinical score on sustained attention tasks.^22^ Furthermore, impairments in executive functions in children with SCT have been linked to increased externalizing behavior problems,^23^ increased social difficulties^24^ as well as increased symptoms of ASD,^25^ psychotic symptoms such as disorganized thought,^26^ and ADHD symptoms.^9^ It is thus not surprising that parents frequently mention that their child's executive dysfunction, amongst others, is a major barrier to learning and academic development.^27^

Previous studies that examined executive functions in the SCT population included school‐aged children, adolescents, and adults. There have been very limited systematic studies on executive functions in early childhood, specifically before 6 years of age and prior to starting the early school years. However, the preschool period (the period between 3 and 6 years of age) is of particular interest when it comes to executive functions, given its development accelerates in the preschool years.^28^ This acceleration is partly due to increased connectivity between neural networks in the brain within this period,^29^ as well as changes at the contextual level (such as social experience^30^) and other cognitive abilities (increasing memory capacity, increasing language abilities and accelerated information processing^31^). Studying this important window in child development in individuals with SCT may help to understand the impact of an extra X or Y chromosome on the developing brain. Differences with typically developing peers are to be expected, given that a high density of genes on the sex chromosomes are essential for brain development,^5^ putting children with SCT at increased risk for neurodevelopmental problems including impairments across executive functions. Also, early identification of these difficulties may reveal risk markers in the development of children with an extra X or Y chromosome, that could prove helpful in identifying targets for early intervention to improve outcomes later in life.

Assessment of executive functions usually relies on a combination of a direct assessment of information processing skills as well as structured behavioral observations in daily life. There is growing evidence that executive functions represent diverse but also united constructs in early childhood.^32^, ^33^ This has also led to new techniques to measure executive functions in young children, such as the Minnesota Executive Function Scale (MEFS App™) that provides a standardized performance‐based assessment of global executive function skills, designed for children ages 2 and up.^34^ It integrates three basic executive functions (working memory, inhibitory control, and cognitive flexibility) into a single graded scale. Because the assessment is sensitive to age and performance, following an adaptive testing protocol, it provides the opportunity to assess and follow to the development of emerging executive functions; with *emerging* meaning still‐developing, not yet stable.^35^ In addition to the neurocognitive assessment of executive functions, structured observations of behavioral problems in daily life are also crucial. Parents are vital observants in providing information on the behavior of their child to gain insight in the developing functions. To illustrate, a child that has difficulties with cognitive flexibility may experience difficulties with a changing caregiver or shift in routine. Using standardized parental rating systems is a well‐accepted evidence‐based method in the assessment of social, emotional, and behavioral functioning.^36^ In this study, both neurocognitive tasks and structured observations are used to provide information on executive functions in young children with SCT.

The current study is, to the best of our knowledge, the first to examine emerging executive functions in a large, international cohort of children with SCT between the ages of 3–7 years old, compared to population‐based controls. As the three trisomy karyotypes (i.e., XXX, XXY, XYY) are characterized by similar neurocognitive impairments during childhood,^19^, ^37^, ^38^ we grouped them into a single sex chromosome trisomy group. The primary goal of the current study was to investigate how executive functions present across different ages in young children with SCT, expressed in terms of information processing skills as well as behavioral observations. Given that executive functions in early childhood are considered a unitary construct, we examined executive functions by using a single performance measure that is appropriate for a large age‐span. In addition, a verbal fluency task was used to examine verbal executive functions specifically. This task was chosen as the language domain is an evident vulnerability in children with SCT,^39^ already at a young age,^40^, ^41^ and we wanted to examine emerging executive functions in the context of both verbal and non‐verbal based information processing. Furthermore, the behavioral report allowed for examination of smaller subdomains of executive functions that could inform on specific vulnerabilities of young children with SCT. Based on earlier research with older children and adults, we hypothesized that even pre‐school age children with SCT already experience difficulties with executive functions.

## 2 MATERIALS AND METHODS

### 2.1 Participants

The current study is part of a large ongoing international longitudinal study (the TRIXY Early Childhood Study, at Leiden University in the Netherlands, including research sites in the Netherlands and the United States of America [USA]). The TRIXY Early Childhood Study investigates the social, emotional, and behavioral development of young children with a trisomy of the X/Y chromosomes (TRIXY). Prior studies from the TRIXY project has been published elsewhere (see e.g., References [37, 38, 40]). For the current study, children aged 3 up to and including 7 years (at baseline) were included. Children were recruited from two sites: The Trisomy of the X and Y chromosomes (TRIXY) Center of Expertise in the Netherlands that recruited children from all Dutch‐speaking countries in Western Europe (*n* = 39) and the eXtraordinarY Kids Clinic in Developmental Pediatrics at Children's Hospital Colorado (CHC) in Denver that recruited children from across the United States of America (*n* = 33). The two clinical groups did not differ in terms of gender distribution (*χ* ^2^ [1,72] = 1.346*, p* = 0.246) nor educational level of caregivers (*p* = 0.224), but differed with respect to age with the American SCT group being on average younger than the Dutch SCT group (*t*[66] = 4.486, *p* < 0.001). Children with SCT were recruited with the help of clinical genetics departments, pediatricians, and national advocacy or support groups for (parents of) individuals with SCT by using recruitment flyers and postings on the internet and social media. Three different recruitment strategies were identified for the SCT group (see Table 1): (a) ‘information seeking parents’, (b) ‘active prospective follow‐up’, and (c) ‘clinically referred cases’. Children from the control group were recruited from day care centers, public institutions, and elementary schools from the western part of the Netherlands by using recruitment flyers.

**TABLE 1:** Demographic variables of the SCT group and the control group

	All ages			3‐ to 4‐year‐olds			5‐ to 7‐year‐olds		
	SCT	Controls	*p*	SCT	Controls	*p*	SCT	Controls	*p*
	*n* = 72	*n* = 70		*n =* 41	*n =* 44		*n =* 31	*n =* 26	
Age in years—*M* (SD)	4.83 (1.29)	4.52 (0.99)	0.120	3.85 (0.61)	3.91 (0.58)	0.935	6.12 (0.75)	5.58 (0.37)	**0.005**
Gender	M = 45	M = 31	**0.030**	M = 28	M = 20	**0.034**	M = 17	M = 11	0.346
	F = 27	F = 39		F = 13	F = 24		F = 14	F = 15	
Parental education level^a^ median (range)	6 (4–7)	6 (2–7)	0.586	6.5 (4–7)	6 (2–7)	0.461	6 (4–7)	6 (2–7)	0.965
Karyotype		N/A			N/A			N/A	
XXX	27			13			14		
XXY	30			20			10		
XYY	15			8			7		
Recruitment strategy (*n*)		N/A			N/A			N/A	
Information‐seeking parents	31			17			14		
Prospective follow‐up	23			13			10		
Clinically referred	18			11			7		
Recruitment site (*n*)									
The Netherlands	39	70		15	44		24	26	
Denver, USA	33			26			7		

*Note:* Significant values are indicated in **bold**.

Abbreviations: F, female; M, male; SCT, sex chromosome trisomies.

^a^ Data from two primary caregivers (1 SCT, 1 control) was missing due to non‐completion of questionnaires.

In total, 72 children with SCT and 70 age‐matched controls from the general population participated in this study with their primary caregiver. The SCT group consisted of 27 girls with 47, XXX, 30 boys with 47, XXY, and 15 boys with 47, XYY. As for the timing of SCT diagnosis, 40 children (56%) had a prenatal diagnosis (i.e., because of [routine] prenatal screening, abnormal ultrasound findings, or advanced maternal age) and 32 children (44%) had a postnatal diagnosis (i.e., because of developmental delay, physical and/or growth problems, or medical concerns). Confirmation of trisomy in at least 80% of the cells was provided by standard karyotyping. Parents were asked to present a copy of the karyotyping report of the child that was provided by their clinician at time of diagnosis. Children from the control group were not subjected to genetic screening. Given the low prevalence of SCT (~1 in 1000) in the general population, we decided that the burn of blood draw for testing for SCT in our control group outweighed their potential utility. We reviewed the possible risk of having a child with undiagnosed SCT in our control group minimal and acceptable. The majority of the children with 47, XXY (57%, *n* = 17) did not receive testosterone replacement therapy at any given time in their development. Parental education level was assessed according to the Hollingshead criteria and ranged from category 1 (no formal education) to 7 (graduate professional training).^42^ When the child was raised by two parents (95%), educational level was averaged over both parents. Parental education level varied from 4 to 7 (median 6) in the SCT group and from 2 to 7 (median 5) in control group. All participants were Dutch‐ or English‐speaking. Children had no history of traumatic brain injury, severely impaired hearing or sight, or colorblindness.

To examine the developmental impact of SCT, children were divided into two age groups: 3‐ to 4‐year‐olds and 5‐ to 7‐year‐olds (see Table 1 for demographic variables). Groups were split at the age of 5 to ensure equal‐enough sample sizes in order to maximize statistical power. This split also optimized the available data regarding the questionnaire data (e.g., 3‐ to 4‐year‐olds filled out a different version of the BRIEF compared to the 5‐to‐7‐year‐olds). The two age‐groups were similar with respect to distribution of karyotype (*χ* ^2^ [2,72] = 2.088, *p* = 0.352) and recruitment strategy (*χ* ^2^ [2,72] = 0.185, *p* = 0.912). Differences between research groups (SCT vs. controls) were investigated within the two age‐groups in terms of age, gender, and parental education level. Within the 3‐ to 4‐year‐old group, the SCT group included significantly more boys but was similar to the control group with respect to age and parental education level. In the 5‐ to 7‐year‐old group, children with SCT were significantly older than controls but groups were similar in terms of gender and parental education level.

### 2.2 Ethics and procedure

This study was approved by the Ethical Committee of the Leiden University Medical Center, Leiden, the Netherlands, and the Colorado Multiple Institutional Review Board in the United States. A team of researchers, consisting of child psychologists, research associates, and graduate students, were trained and supervised by professionals in the field of child psychology, certified and specialized in neuropsychological assessment. All primary caregivers signed a written informed consent prior to assessment. Children were tested either in a quiet room at the University (SCT: 53%, controls: 43%) or at home (SCT: 47%, controls: 57%) using written protocols detailing all procedures and verbal instructions to standardize assessments. Researchers from Leiden University were responsible for project and data‐management (i.e., training and supervision of researchers, processing, and scoring of data). The primary caregiver (92% female) of the child completed the questionnaire in either Dutch or English using the online survey software Qualtrics (http://www.qualtrics.com/).

### 2.3 Instruments

#### 2.3.1 Executive function skills

**FIGURE 1:**
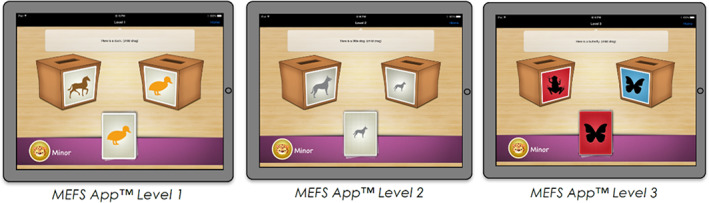
First three levels of the MEFS App™. Children are instructed to sort cards into boxes based on a specific rule that increases in difficulty when a child progresses through the levels (displayed here: 1: horses versus ducks, 2: large versus small, 3: red versus blue). *Source*: Pictures from ‘Minnesota Executive Function Scale App™ and Admin Portal User Guide’ (p. 4) by Carlson and Zelazo,^66^ Reflection Sciences, Inc.™, St. Paul, MN. Reprinted with permission.

Global executive function skills were measured with the Minnesota Executive Function Scale (MEFS App™): a standardized performance‐based assessment of global executive function skills, designed for children ages 2 and up, that is administrated on a touch‐screen tablet.^34^ Administration time is usually 2–6 min (average of 4 min). The reliability and validity are high and the app has been used in general and clinical populations.^34^ The MEFS App™ is a comprehensive executive function measure that goes down to 2 years of age and spans throughout adulthood and provides a single graded scale based on the combined assessment of working memory, inhibitory control, and cognitive flexibility. The MEFS assessment has increasing difficulty and is sensitive to age and performance, according to an adaptive testing protocol based on the responses of the child. Children are asked to sort cards into two boxes according to one rule and then switch to sorting the same cards again using an opposite or conflicting rule (see Figure 1). It requires children to switch between rules and inhibit one's automatic response. Furthermore, working memory is required to remember the current rule(s) for each trail. Because of its adaptive testing protocol, the MEFS provides a sensitive assessment of each individual child and his/her global executive function skills. After finishing the task, a total score (0–100) is calculated based on an algorithm that takes both accuracy and response time into account, with higher scores reflecting better executive function skills. In analyses, either raw or standardized scores were used (see Section 2.4). Standardized scores were calculated differently depending on the recruitment site. For children from the Netherlands, scores from the current control group were used to calculate standardized scores (percentile scores). For children from the United States, scores from the general population were provided by the MEFS‐app and converted into standardized scores (percentile scores).

#### 2.3.2 Verbal executive function skills

To assess verbal executive function skills a measure of verbal fluency was used. Verbal fluency is commonly described as a measure of executive function in the context of verbal information^43^ because it requires goal‐directed behaviors such as cognitive flexibility, strategic planning, and error‐monitoring.^14^ For this study, the subtest ‘word generation’ of the NEPSY‐II Developmental Neuropsychological Assessment was used.^44^ In this subtest children are asked to generate words within two specific categories (‘animals’ and ‘food/beverages’) as many as possible within a 60‐s period for each category. Administrated answers were afterwards coded to yield either 0 points for an incorrect answer or 1 point for a correct unique answer. Higher scores represent higher levels of verbal fluency. Either summed raw scores or scaled scores were used in analyses (see Section 2.4). Scaled scores were derived from the manual, using the appropriate norm group depending on the language spoken by the child (Dutch or English).

#### 2.3.3 Executive functions in daily behaviors

The Behavior Rating Inventory of Executive Function (BRIEF) was used as an assessment tool of everyday executive functions.^45^, ^46^ It is developed to capture real‐world manifestations of executive dysfunction, by focusing on children's everyday behaviors at home^45^, ^46^ For the current study, primary caregivers completed either the BRIEF‐P for a child aged 3; 0–4; 11 years (*n =* 84) or the BRIEF school‐age for a child aged between 5; 0 and 6; 11 years (*n* = 54). A small subset of children (aged 5; 0–5; 11) fell within the appropriate age‐range of both questionnaires. For future follow‐up purposes caregivers of these children completed the BRIEF school‐age. Both questionnaires in the Dutch and US version have satisfactory internal consistency (ranging from 0.78 to 0.98), test–retest reliability (ranging from 0.72 to 0.90) and convergent and discriminant validity (for the exact values see the appropriate manuals; US^45^, ^46^; NL^47^, ^48^). The BRIEF‐P and BRIEF school‐age comprise 63‐item and 86‐item rating scales respectively, with a 3‐point rating including never, sometimes, and often. Both questionnaires have a total score (Global Executive Composite: GEC) with two or three indices, subdivided into multiple subscales. The overlapping subscales present in both BRIEF questionnaire versions include: Inhibit, Shift, Emotional Control, Working Memory, and Planning and Organization. Additional subscales for the BRIEF school age were Monitor, Organization of Materials, and Initiate. To compare all children across ages independently of which BRIEF version was administrated, for each child total scores were divided by the specific number of items to create mean scores. Higher scores indicate more difficulties. Either summed raw scores or standardized scores (*T*‐scores) were used in analyses (see Section 2.4). Standardized scores were derived from the BRIEF(‐P) manual, using the appropriate norm group depending on the recruitment site (The Netherlands or USA).

#### 2.3.4 Intellectual functioning

To control for potential group differences due to overall differences in intelligence, full scale IQ was estimated with a shortened version of the Wechsler Preschool and Primary Scale of Intelligence—third edition (English version: WPPSI‐III^49^; Dutch version: WPPSI‐III‐NL^50^). This short version with four subtests (Vocabulary, Similarities, Block Design, and Matrix Reasoning) has been found to provide a valid and good estimation of the full‐scale intelligent quotient.^51^ Standard IQ‐scores were derived from manual, using the appropriate norm group depending on the recruitment site (The Netherlands or USA).

### 2.4 Statistical analyses

Some data were missing due to outliers or technical dysfunction. Resulting from this, sample sizes varied from 60 to 72 SCT children and 67 to 69 controls per analysis. Data were analyzed using IBM SPSS version 25. Demographic characteristics were compared with independent sample *t*‐tests and Chi‐square tests. As preliminary analysis, to examine whether recruitment site was relevant to executive function outcomes, *t*‐tests were used to examine mean group differences within the SCT group (Dutch vs. US) and mean group differences within the control group (Dutch or US‐referenced norms).

For each executive function measure, the following analyses steps were taken. First, to test the hypothesis that executive functions were dependent on age, a linear approach using correlation analyses was used to maximize statistical power. Because raw scores were used in these analyses, they needed to be corrected for recruitment site. We used PROCESS, a bootstrapping, nonparametric resampling procedure (for further information see References [52, 53]), to control for the potential role of recruitment site in the SCT group. Subsequently, if significant effects of age on EF measures were found, subsequent ANOVAs per age‐group were performed to identify group differences at specific ages using standardized norms of the EF measures. These ANOVAs were carried out as post‐hoc tests to analyze the differences between SCT and control within the different age‐groups (3‐ to 4‐year‐olds and 5‐ to 7‐year‐olds) and thus only included those variables that were found significantly related to age. Third, a correlation analysis with the standardized executive function measures (MEFS, NEPSY, BRIEF) and FSIQ was performed to examine the influence of IQ on the results. If significance was revealed, post‐hoc analyses were performed using ANOVAs to determine the specific role of IQ in executive functions.

Finally, two MANOVA's were performed to compare executive function measure outcomes (dependent variables, standardized scores) for the influence of recruitment strategy and karyotype, as a marker of data quality and representivity for the entire SCT group.

For correlation analyses with age, Pearson's product moment correlation coefficient was used. Level of significance was set at *p* = 0.05. For all significant effects, Cohen's *d* addressed effect size (0.2 = small effect; 0.5 = medium effect; 0.8 = strong effect^54^).

## 3 RESULTS

### 3.1 Preliminary analyses

Preliminary analyses revealed minimal evidence of site effects on executive function scores (see Appendix A for the exact results). Within the SCT group, Dutch and American children did not differ on executive functions. Further, children in the control group did not differ from normative Dutch and US scores, with the exception of verbal executive function scores on the NEPSY subtask. Taken together these results provide the support to pool SCT children together and treat them as a singular group of SCT children and that controls are representative controls for both clinical samples.

### 3.2 Role of age in executive functions

The results from the correlation analysis between the variable ‘age’ and executive function parameters are shown in Table 2. Recruitment site was included as a covariate in the analyses but there were no significant interaction effects (see Appendix B and Table B1 for the exact results), thus indicating that recruitment site (The Netherlands or United States) did not influence the results.

**TABLE 2:** Correlations between age and executive functions for the SCT and control group

	SCT age	CG age	Fisher *r* to *z* transformation	
	*r*	*r*	*z*	*p*
1. Global executive function skills (MEFS)	0.590***	0.764***	−1.810	**0.035**
2. Verbal executive function skills (NEPSY)	0.728***	0.702***	0.294	0.384
3. Overall executive functioning (BRIEF GEC)	0.418**	0.265*	0.998	0.159
4. Inhibit (BRIEF)	0.195	0.104	0.535	0.296
5. Shift (BRIEF)	0.294	0.100	1.164	0.122
6. Emotional control (BRIEF)	0.455**	0.312**	0.966	0.167
7. Working memory (BRIEF)	0.422**	0.154	1.694	**0.045**
8. Plan and organize (BRIEF)	0.128	−0.325**	2.677	**0.004**

*Note*: Higher scores reflect better performance for MEFS and NEPSY assessments, lower scores on the BRIEF reflect better functioning. Raw scores were used in analyses; in the SCT group, recruitment site was included as covariate. Significant values are indicated in **bold**.

Abbreviations: BRIEF, Behavior Rating Inventory of Executive Function; CG, control group; GEC, general executive composite; MEFS, Minnesota Executive Function Scale; NEPSY, NEPSY‐II Developmental Neuropsychological Assessment; SCT, sex chromosome trisomy.

* *p* < 0.05;

** *p* < 0.01;

*** *p* < 0.001.

**FIGURE 2:**
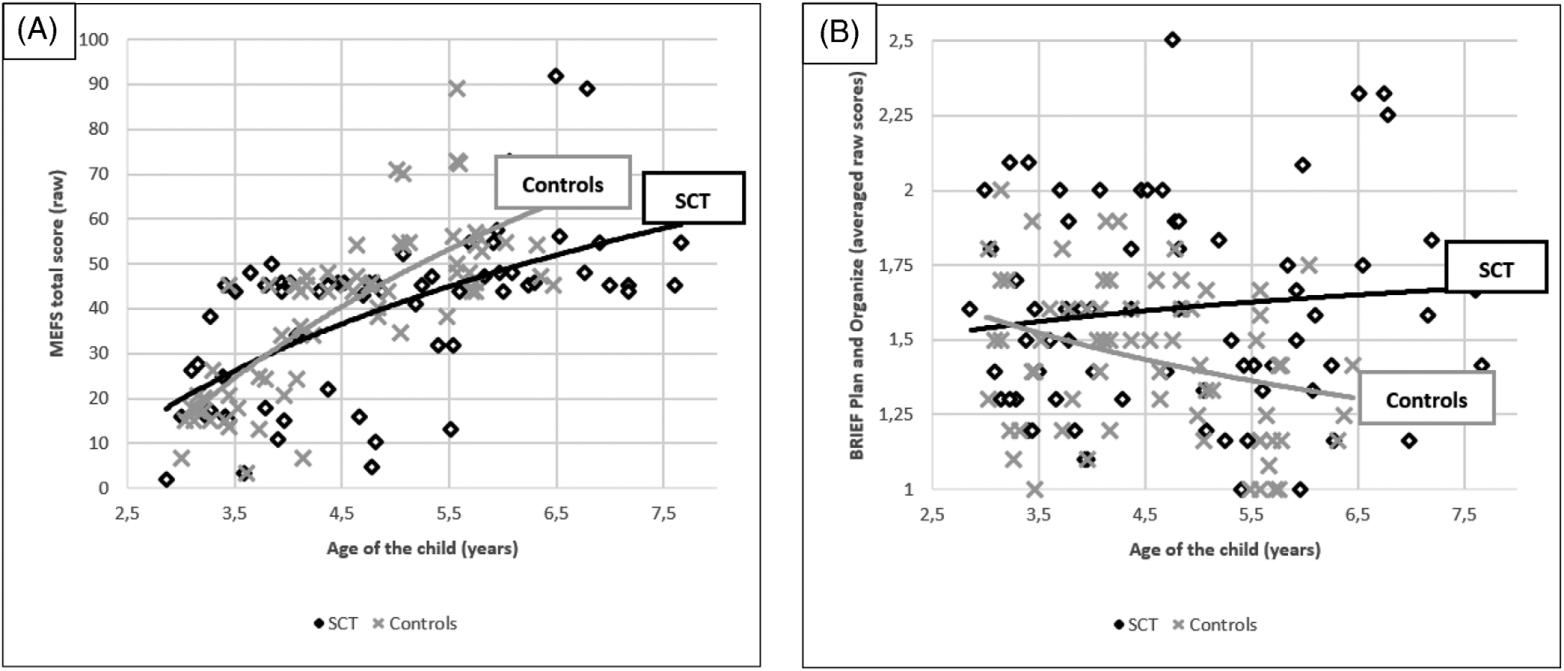
Scatter plot for child's age against executive function scores (A) global executive function skills (MEFS) and (B) daily life plan and organize problems (BRIEF)) for individuals with SCT and controls, with correlation lines for each group displayed. Higher scores reflect better performance for MEFS assessment (A), lower scores on the BRIEF (B) reflect better functioning.

Within the SCT group, there were significant correlations between age and global executive function skills, verbal executive function skills, and different aspects of executive functions in daily life including emotional control and working memory. Most, but not all, correlations were also significant in the control group. For global executive function skills, working memory, and plan and organizing, the strengths of correlations differed significantly between research groups (see Table 2). For the other domains, the strength of correlations did not differ significantly between the SCT and control group. These results indicate that age is an important factor in executive function (problems), with differential presentation across ages, as is visible in Figure 2.

### 3.3 Age‐specific group differences in executive functions

As age appeared to be associated with executive functions and showed differential patterns across ages and domains (for an illustration of these specific relations see Figure 2), specific age‐groups (3–4 year‐olds and 5‐ to 7‐year‐olds) were examined to identify impairments in executive functions at specific ages.

#### 3.3.1 3‐ to 4‐year‐olds

There were significant group differences found for executive functions between 3‐ to 4‐year‐old children with SCT and controls (see Table 3). Children with SCT had significantly lower verbal executive function skills than the control group (medium effect size). For daily life behavior, parents of 3‐ to 4‐year‐old children with SCT reported, on average, more executive function difficulties in daily life compared to controls, also with great variability. A significant group difference was found for working memory with medium effect size. Worth noting is that the domain of emotional control was trend significant (*p* = 0.067), with a medium effect size, as well as total general daily life difficulties (*p* = 0.066). With regards to global executive function skills assessed with a task, there was no significant group difference between the SCT and control group (*p* = 0.511).

**TABLE 3:** Age‐specific differences between research groups on executive functions

	3‐ to 4‐year‐olds					5‐ to 7‐year‐olds				
	SCT	Controls	Statistical test results			SCT	Controls	Statistical test results		
	*M* (SD)	*M* (SD)	*F*	*p*	*d*	*M* (SD)	*M* (SD)	*F*	*p*	*d*
Neurocognitive skills										
Global executive function skills (MEFS)	39.54 (24.40)	35.89 (25.31)	0.436	0.511	‐	58.93 (19.47)	74.80 (14.84)	10.371	**0.002**	0.9
Verbal executive function skills (NEPSY)	10.59 (2.21)	11.86 (2.36)	5.555	**0.021**	0.5	8.71 (3.09)	11.31 (2.78)	10.449	**0.002**	0.9
Overall executive functions (BRIEF GEC)										
Total daily life executive functioning	55.02 (11.75)	50.77 (9.07)	4.608	0.066	0.4	54.29 (11.99)	44.35 (9.64)	11.592	**0.001**	0.9
Domains of executive functions (BRIEF indices)										
Inhibitory‐self‐control	52.66 (11.37)	49.81 (8.54)	1.691	0.197	‐	53.40 (14.17)	43.65 (10.65)	8.255	**0.006**	0.8
(Emergent) Metacognition	56.15 (11.34)	51.35 (9.49)	4.439	**0.038**	0.5	55.33 (13.02)	45.31 (9.14)	10.787	**0.002**	0.9
Flexibility	54.27 (12.45)	50.49 (9.44)	2.475	0.120	‐	N/A	N/A			
Behavioral specific executive functions (BRIEF subscales)										
Emotional control	53.56 (12.33)	49.30 (8.44)	3.441	0.067	0.4	56.55 (12.83)	44.85 (9.80)	14.512	**0.001**	1.0
Working memory	56.95 (11.53)	51.09 (10.13)	6.134	**0.015**	0.5	56.38 (11.26)	46.27 (9.34)	13.304	**0.001**	1.0
Plan and organize	54.10 (11.29)	51.63 (8.30)	1.313	0.255	‐	50.58 (10.59)	43.62 (6.68)	8.423	**0.005**	0.8
Initiate (only 5+)	N/A	N/A				53.10 (11.22)	47.15 (8.04)	4.928	**0.031**	0.6
Organization of materials (only 5+)	N/A	N/A				52.33 (12.83)	48.96 (9.51)	1.216	0.275	‐
Monitor (only 5+)	N/A	N/A				49.68 (10.35)	45.54 (8.26)	2.709	0.105	‐

*Note*: Higher scores reflect better performance for MEFS and NEPSY assessments, lower scores on the BRIEF reflect better functioning. Different standardized scores are used for each measure: MEFS included percentile scores; NEPSY included scaled scores with a mean of 10, SD of 3; BRIEF included T‐scores with a mean of 50, SD of 10. Significant values are indicated in **bold** **.**

Abbreviations: BRIEF, Behavior Rating Inventory of Executive Function; CG, control group; GEC, general executive composite; MEFS, Minnesota Executive Function Scale; NEPSY, NEPSY‐II Developmental Neuropsychological Assessment; SCT, sex chromosome trisomy.

#### 3.3.2 5‐ to 7‐year‐olds

There were significant group differences for executive functions between 5‐ to 7‐year‐old children with SCT and controls (for results see Table 3). On the neurocognitive tasks, children with SCT had significantly lower global executive function skills and lower verbal executive function skills compared to controls, with large effect sizes. For executive function difficulties in daily life behavior, parents of 5‐ to 7‐year‐old children with SCT reported, on average, more executive function difficulties in daily life, compared to controls, with difficulties on almost all behavioral domains. Effect sizes range from *medium* (*d* = 0.6 for Initiate and Monitor) to *large* (*d* = 1.0 for Working Memory). Worth noting is the greater variance in scores in the SCT group on almost all BRIEF subdomains compared to controls.

### 3.4 Role of IQ in executive functions

Results from the independent samples *t*‐test showed that children with SCT had a significantly lower full‐scale IQ (*M* = 95.51, SD = 19.75) than controls (*M* = 109.31, SD = 13.242), *t*(115) = −4.752, *p* < 0.001. Within the SCT group, no significant correlation was found between IQ and global executive function skills (*r* = −0.084, *p* = 0.514). In contrast, IQ was significantly associated with verbal executive function skills (*r* = 0.497, *p* < 0.001) and daily life executive functions (BRIEF GEC score and all five BRIEF subscales; significant *r*‐values ranging from −0.299 to −0.271).

To evaluate the relevance of these findings, we reran previous analyses within separate IQ‐groups (children with SCT and below average IQ vs. children with SCT and average IQ) as compared to controls (see Table 4 for the results). In both IQ groups, significant group differences were found between SCT and controls on almost all parameters, showing that difficulties with executive function were found across the range of intelligence levels and were not limited only to those children with below average IQ (also see Appendix C for additional analysis).

**TABLE 4:** Group differences between controls and SCT children across IQ‐levels

	Controls	SCT		Statistical test results			
	*N* = 67	IQ < 85 *N* = 17	IQ > 85 *N* = 50	Controls vs. SCT IQ < 85		Controls vs. SCT IQ > 85	
	*M* (SD)	*M* (SD)	*M* (SD)	*F*	*d*	*F*	*d*
Verbal executive function skills (NEPSY)	11.71 (2.53)	7.46 (3.55)	10.39 (2.22)	27.239***	1.4	8.173***	0.6
Overall executive functions							
General executive composite (GEC)	48.21 (9.84)	56.88 (10.68)	52.74 (11.80)	10.177***	0.8	5.134**	0.4
Behavioral specific executive functions							
Emotional control scale	47.36 (9.10)	55.94 (11.75)	53.66 (12.95)	10.676***	1.1	9.690***	0.6
Working memory scale	49.27 (10.19)	57.94 (12.28)	55.26 (10.98)	9.021***	1.1	9.79***	0.6
Plan and organize scale	48.84 (8.58)	54.65 (9.45)	51.24 (8.53)	5.974**	0.9	1.948	‐

*Note*: Level of significance: *p < 0.1; **p < 0.05; *** *p* < 0.01.

Abbreviations: SCT, sex chromosome trisomy; CG, control group; below‐average IQ: estimated full‐scale intelligence quotient below 85; average IQ: estimated full‐scale intelligence quotient above 85.

### 3.5 Role of karyotype and recruitment strategy

In terms of the comparability of karyotypes, there were no significant group differences found between the three different karyotypes on executive functions (Pillai's trace = 0.160, *F*[6,104] = 1.507, *p* = 0.183). Which karyotype a child carried did not appear to affect the degree of executive functions, both in neurocognitive performance or in daily life behavior. Finally, to examine whether ascertainment method was relevant to the increased risk for executive function difficulties in children with SCT, a MANOVA was performed with executive functions (MEFS total score, NEPSY total score, BRIEF General Executive Composite score) as dependent variables and recruitment strategy within the SCT group (prospective follow‐up, information seeking parents, clinically referred cases) as independent variable. There was no effect of ascertainment on executive functions (Pillai's trace = 0.190, *F*[3,104] = 1.822, *p* = 0.102): how children enrolled in the study did not appear to affect the degree of executive functions, both in neurocognitive assessment as in daily life behavior.

## 4 DISCUSSION

The present study investigated emerging executive functions in young children with SCT compared to population‐based controls. We assessed whether children with SCT already show an increased risk for executive function difficulties at a young age. Core to the study was the inclusion of a large international group of children with an extra X or Y chromosome between the ages of 3 and 7 years of which the majority had a prenatal diagnosis, which could provide insight in the developmental impact of SCT from a prospective point of view. Also critical to discussion of results is the acknowledgement of the variability in the SCT group and marked overlap with the control group such that many participants in the SCT group had scores that were similar or even improved compared to some individuals in the control group. However, statistical analyses of group differences are important as they help to delineate specific domains affected by SCT in order to understand how to support and to develop treatments for the proportion of individuals with SCT whose challenges in these areas are clinically significant such that they affect daily functioning and quality of life.

Our results revealed that children with SCT are at increased risk for problems with emerging executive functions, from as early as 3 years old, and that those problems appear more pronounced at an older age. Furthermore, impairments in executive functions appear broader than the language domain alone, extending to other areas as well, suggesting that impaired executive functions are part of the SCT neurodevelopmental profile, even when intelligence levels are in the typical range. To illustrate, specific difficulties are found for 3‐ to 4‐year‐old children with SCT in the area of verbal fluency and working memory. Children with SCT aged 5–7 years experienced more and broader executive function impairments than their peers, showing difficulties with global executive functions, verbal fluency, cognitive flexibility, emotional control, working memory, and planning and organizing. This is the first study showing that there is a developmental impact of SCT on emerging executive functions before the age of 7 years and that children with SCT are at significant risk for difficulties with executive functions in early childhood. Our findings add to the already existing literature done with older participants.^9^, ^18^, ^20^, ^21^, ^23^

The increased risk for emerging executive function difficulties in children with SCT indicates that their ability to show purposeful, goal‐directed, and problem‐solving behavior is affected, from as early as 3 years old. The impact for these children is significant, given that preschool executive functions are vital for school readiness,^55^ putting children with SCT at a substantial disadvantage at school entry. Furthermore, executive functions continue to be an important factor throughout childhood with regards to academic success, given that early executive functions also predict math and reading competence.^56^ Next to school readiness and academic success, adequate executive functions also impact psychological well‐being, considering that impairments in executive functions has been linked to various symptoms of psychopathology in the general population, including both internalizing and externalizing behavioral problems.^57^ Social, emotional, and behavioral problems are frequently reported in the SCT population^4^, ^58^, ^59^, ^60^, ^67^ and our results suggest that emerging executive functions could be one of the key components in explaining the variability as well as the increased risk for psychopathology in this genetically at‐risk group. Previous studies using older samples have already provided some evidence for this hypothesis showing a link between impairments in executive functions and social–emotional and behavioral problems,^24^ psychotic symptoms,^26^ and ADHD symptoms.^9^ Future studies should further investigate the relationship, both cross‐sectionally as well as longitudinally, between emerging executive functions and psychological functioning in this young population of SCT.

Our study results also underline the importance of a developmental approach with regards to neurocognition in early childhood. Albeit we studied these children cross‐sectionally, our results showed that increasing age is associated with more prominent and broader executive function difficulties in children with SCT. Deviations from controls were already evident from 3 years of age, but children in the 5‐to‐7‐year‐group showed more pronounced executive function difficulties (as illustrated by larger effect sizes) that appeared across multiple areas of functioning. Existing literature on the relation between age and executive functions in SCT is limited. However, our findings nicely complement one other study examining age‐dependent effects of daily life executive functions,^18^ in which children with Down syndrome and Klinefelter syndrome between the ages of 5–18 years old were compared to typically developing peers. The results from this study also showed more pronounced executive function difficulties with increasing age in the group children with an extra X chromosome, specifically in two areas of executive functions: plan/organize and initiate. Taken together, these findings suggest that the vulnerability for executive function difficulties in SCT might be already present from a young age but may not be limited to early childhood and is suggested to continue into later development. Looking from a neuropsychological perspective, we see a genetically at‐risk group of children who show a differential pace in emerging neurocognitive functions, which could point to a suboptimal maturation of the brain and thereby possibly implicating future neurodevelopment. Albeit that our results show that a developmental approach provides additional insight into the impact of SCT, we acknowledge that our results were examined within a cross‐sectional design and encourage the study of developmental trajectories of children with SCT using longitudinal studies to add validity to these results.

Within the age‐specific executive function profiles, our finding on emotional control difficulties is worth highlighting. These results indicate that emotional control might be a relevant at‐risk marker for young children with SCT, given that difficulties in this area are present at a young age (albeit trend significant at age 3) and appears to be one of the most pronounced weaknesses for children with SCT between the ages of 5 and 7 years. *Emotional control* represents an individual's ability to modulate emotional responses. Poor emotional control can be expressed as emotional liability or emotional explosiveness and caregivers usually describe these children as having overblown emotional reactions to seemingly minor events. From a developmental perspective, difficulties with adequately regulating and controlling your emotions have been linked to higher levels of social, emotional, and behavioral problems,^61^ highlighting the importance of emotion regulation abilities for quality of life. The findings of the current study complements existing literature who also described significant difficulties in emotional control and regulation in adolescents and adults with SCT, expressed in behavioral problems^18^ as well as physiological regulation difficulties.^62^ Moving forward, now that we have established that children with SCT are at increased risk for emotion regulation difficulties from an early age on, it is worthwhile to examine its developmental trajectory using a diverse set of measures, including those of the affective arousal system. These findings could provide further insight on the predictive value of emotion regulation difficulties in early childhood for later development, and also point to emotional control as a target for early treatment programs.

Important to note is the broad variation in executive functions observed between children with SCT in the current study. For clinical care, it is imperative to realize that having a specific genetic variation does not reliably predict what the exact outcome will be for any given individual. Thus, working in a clinical setting with children with SCT, professionals need to be aware of the variation in executive functions between children with SCT just as much as the developmental risk for impaired executive functions. From a young age, difficulties with (emerging) executive functions could be part of an individual's neurocognitive profile, even in the face of a typical intelligence, and therefore requires specific attention in assessment using age‐appropriate and valid measures (including but not limited to neurocognitive tests, structured observations, development history interview). Identifying impairments in (specific areas of) executive functions can result in specific guidelines on what function needs to be supported during treatment. Given the significant relevance of executive functions on many developmental outcomes in childhood, specifically school readiness and achievement,^63^ it is important to consider support options for preschool children with SCT who already experience difficulties in this area. Up to 48% of young children with SCT already receive early childhood intervention services before the age of 6, including preschool academic support,^68^ in which the area of executive functions could be addressed as well. Treatment and/or support could include training the specific executive function skill, using stronger‐developed skills to compensate for the less‐developed executive function, and/or adjusting the context to the limitations or the dysfunction itself by implementing tools or lowering expectations. Empirical studies on executive function intervention in children with SCT are non‐existent, but the study of effect of executive function interventions in the general population is a promising, but emerging field.^69^ A recent meta‐analytic review on the effectiveness of cognitive training in preschoolers^64^ showed that there is an overall effect of cognitive training in improving executive functions, especially in at‐risk groups (ADHD or children with low socio‐economic status), suggesting that those at risk might benefit more from stimulation than children without additional risks. However, Scionti et al.^64^ did not find an effect of cognitive training on additional outcomes, such as psychological or behavioral benefits. In sum, while these results indicate that executive function training might also be a valuable component in treating (emerging) executive function difficulties in children with SCT, it is crucial not to focus narrowly on improving executive functions alone, but also address the social, emotional, and behavioral development in addition to the social context in which a child with SCT grows up in (family and school).

While the results of the current study are promising and the size of the sample is noteworthy, especially since genetic population are difficult to recruit, the current study also has limitations that should be addressed. As mentioned previously, our results are based on a cross‐sectional designed study. Longitudinal studies are crucial to add validity to our age‐dependent results and could provide further insight in the developmental pathways of EF in children with SCT. Also, by collapsing across sex chromosomal trisomies we were not able to assess the specific contribution of karyotype (XXX, XXY, and XYY) on impairments in executive functions. Thirdly, the current study did not examine the effect of early testosterone hormone treatment on the neurocognitive profile in the SCT subgroup with XXY. Although treatment with testosterone might be considered to improve the physical implications of a micropenis, the evidence for potential benefits of early testosterone on (neuro)developmental outcomes in infants with Klinefelter syndrome is still limited.^65^ We support the initiative of Aksglaede et al.^65^ who call for a randomized and placebo‐controlled trial with an adequately powered cohort sample: one of which is currently underway (PI Davis, NCT03325647).

## 5 CONCLUSION

In sum, the present study showed that when it comes to emerging (e.g., still‐developing) executive functions, many (but not all) children with SCT experience reduced performance and everyday functioning, which seems to be present from a young age (3 years). There appears to be a broader and more significantly impaired executive function profile in older children with SCT, suggesting increasing impairments in executive functions with age. These impairments in executive functions are broader than the language domain alone, extending to other areas as well, including planning, emotional control, and working memory. The increased risk for impaired executive functions appears to be robust and present above and beyond differences in intelligence, karyotype, recruitment site, and recruitment strategy. This increased risk in early childhood might point to a suboptimal brain maturation in children with SCT. Additional research is warranted using a larger sample that also examines the predictive value of executive functions in terms psychopathology. Our data indicate that emotional control could be an important candidate. Clinically, the results from the study show that impairments in executive functions are part of the broad variation that can occur in SCT, even in the presence of typical levels of intelligence. It highlights the importance of early monitoring and screening of executive functions in preschool children with SCT, which may allow for preventive and early intervention to optimize developmental outcomes.

## CONFLICT OF INTEREST

The authors declare no conflict of interest.

## Data Availability

The data that support the findings of this study are available from the corresponding author upon reasonable request.

## APPENDIX A

### A.1

See Table A1.

**TABLE A1:** Group differences on executive function parameters (standardized scores) in the SCT and control group.

	SCT NL (*N* = 39)	SCT US (*N* = 33)	*p*	Controls NL‐ref group (*N* = 69)	Controls US‐ref group (*N* = 69)	*p*
	*M* (SD)	*M* (SD)		*M* (SD)	*M* (SD)	
Neurocognitive skills						
Global executive function skills (MEFS)	52.56 (26.13)	41.94 (21.07)	0.079	50.56 (29.36)	46.78 (18.78)	0.373
Verbal executive function skills (NEPSY)	9.97 (3.19)	9.36 (2.16)	0.409	11.65 (2.53)	12.51 (2.55)	0.049
Overall executive functions problems (BRIEF GEC)						
Total daily life executive functioning	53.38 (11.24)	56.27 (12.36)	0.303	48.35 (9.73)	48.79 (10.25)	0.796
Behavioral specific executive functions (BRIEF subscales)						
Inhibit	49.69 (10.13)	51.88 (11.27)	0.389	48.26 (8.83)	49.90 (8.12)	0.258
Shift	53.62 (11.08)	55.18 (13.09)	0.584	48.91 (10.23)	50.14 (10.43)	0.486
Emotional control	54.00 (11.60)	55.85 (13.69)	0.537	47.62 (9.16)	48.20 (9.84)	0.721
Working memory	54.56 (10.96)	59.24 (11.41)	0.081	49.27 (10.05)	51.64 (10.32)	0.174
Plan and organize	52.36 (10.48)	52.85 (11.06)	0.853	48.61 (8.61)	48.81 (7.53)	0.885

*Note*: Different standardized scores are used for each measure: MEFS included percentile scores; NEPSY included scaled scores with a mean of 10, SD of 3; BRIEF included T‐scores with a mean of 50, SD of 10.

Abbreviations: SCT, sex chromosome trisomy; CG, control group; MEFS, Minnesota Executive Function Scale; NEPSY, NEPSY‐II Developmental Neuropsychological Assessment; BRIEF, Behavior Rating Inventory of Executive Function; GEC, General Executive Composite.

## APPENDIX B

### B.1 Moderation effect of recruitment site in the SCT group

*Analysis description*: Bias‐corrected bootstrapping analyses (PROCESS) were conducted to test for a moderating effect of the recruitment site (Dutch or US) on the relations between age and executive function parameters in the SCT group. There were no significant interaction (e.g., moderation) effect of recruitment site, revealing that the relation between age and executive function outcomes did not differ across sites. See Table B1 for the exact results.

**TABLE B1:** PROCESS results on the moderation effect of recruitment site in the SCT group in the relation between age and executive function outcomes.

	Recruitment site (interaction effect)			
	*b*	SE	*t*	*p*
1. Global executive function skills (MEFS)	1.63	1.62	1.00	0.320
2. Verbal executive function skills (NEPSY)	−0.71	0.64	−1.10	0.275
3. Overall executive functioning (BRIEF GEC)	0.03	0.04	0.89	0.379
4. Inhibit (BRIEF)	0.02	0.05	0.34	0.735
5. Shift (BRIEF)	0.02	0.05	0.46	0.650
6. Emotional control (BRIEF)	0.04	0.06	0.76	0.451
7. Working memory (BRIEF)	0.05	0.05	1.05	0.296
8. Plan and organize (BRIEF)	−0.01	0.04	−0.03	0.976

*Note*: Raw scores on all outcome parameters were used in the PROCESS analyses.

## APPENDIX C

### C.1 Role of estimated IQ in the group differences between SCT and controls on executive function outcomes

To address the robustness of our results concerning the role of IQ, we also ran a MANOVA with the executive function parameters as dependent variables, research group as independent variable and included IQ as covariate. These results again showed a multivariate effect of research group (*p* = 0.004), next to a multivariate effect of IQ (*p* < 0.001). Thus, caregivers of children with SCT reported significant more daily life executive functions problems (while controlling for IQ), specifically emotional control (*p* = 0.017) and working memory (*p* = 0.022). Also, children from the SCT group performed significantly less well than controls on verbal executive functions (*p* = 0.017). Group differences were non‐significant for inhibit (*p* = 0.969), shift (*p* = 0.199), and plan/organize (*p* = 0.331).
